# CDK10 in Gastrointestinal Cancers: Dual Roles as a Tumor Suppressor and Oncogene

**DOI:** 10.3389/fonc.2021.655479

**Published:** 2021-06-30

**Authors:** Zainab A. Bazzi, Isabella T. Tai

**Affiliations:** ^1^ Division of Gastroenterology, Department of Medicine, University of British Columbia, Vancouver, BC, Canada; ^2^ Canada’s Michael Smith Genome Sciences Centre, British Columbia (BC) Cancer, Vancouver, BC, Canada

**Keywords:** colorectal cancer, cyclin-dependent kinases, gastrointestinal cancers, hepatocellular carcinoma, gastric cancer, biliary tract cancer, CDK10

## Abstract

Cyclin-dependent kinase 10 (CDK10) is a CDC2-related serine/threonine kinase involved in cellular processes including cell proliferation, transcription regulation and cell cycle regulation. CDK10 has been identified as both a candidate tumor suppressor in hepatocellular carcinoma, biliary tract cancers and gastric cancer, and a candidate oncogene in colorectal cancer (CRC). CDK10 has been shown to be specifically involved in modulating cancer cell proliferation, motility and chemosensitivity. Specifically, in CRC, it may represent a viable biomarker and target for chemoresistance. The development of therapeutics targeting CDK10 has been hindered by lack a specific small molecule inhibitor for CDK10 kinase activity, due to a lack of a high throughput screening assay. Recently, a novel CDK10 kinase activity assay has been developed, which will aid in the development of small molecule inhibitors targeting CDK10 activity. Discovery of a small molecular inhibitor for CDK10 would facilitate further exploration of its biological functions and affirm its candidacy as a therapeutic target, specifically for CRC.

## Introduction

Cyclin-dependent kinases (CDKs) are a family of serine/threonine protein kinases that play a critical role in regulating cellular processes, including cell division and cell death (1). Currently, more than 20 members of the CDK family have been identified by their characteristic ATP-binding pocket, PSTAIRE-like cyclin-binding domain and activating T-loop motif (1, 2). CDKs become active when non-covalently bound to their cyclin partner, *via* association with the PSTAIRE-like cyclin binding domain. The interaction of a CDK to its cyclin partner forms a heterodimer, in which the CDK acts as the catalytic subunit and the cyclin functions as the regulatory subunit. Cyclins are responsible for regulation of a CDK’s kinase activity and substrate specificity. CDKs, their cyclin interacting partners, and functions are summarized in **Table 1**.

**Table 1:** CDKs in gastrointestinal cancers.

Name	Putative Functions	Expression in Tumors vs. Normal Tissue					References
		CRC	Gastric Cancer	Liver Cancer ¥	Pancreatic Cancer	Other	
CDK1	Regulates the G2/M-phase transition	↑	↑	↑	↑		(3–6)
CDK2	Promotes cell cycle G1/S-phase transition	↑	NS*	NS*	NS*		(7)
CDK3	Involved in G0/G1 transition *via* phosphorylation of pRb.	↑	↓*	↓*	↓*		(8, 9)
CDK4	Regulates the G1/S-phase cell cycle transition *via* phosphorylation of Rb	↑	↑	↑	↑		(10–13)
CDK5	No known cell cycle functions Shown to be involved in brain development and neuronal differentiation	↑	↓	↑	↑		(14–18)
CDK6	Regulates the G1/S-phase cell cycle transition *via* phosphorylation of Rb	↑	↑	NS*	NS*	↑esophageal	(19–21)
CDK7	Activates CDK1, CDK2, CDK4 and CDK6 *via* phosphorylation of specific threonine sites; Forms complex with TFIIH to regulate RNA polymerase II transcription	NS*	↑	↑*	↑		(22, 23)
CDK8	Regulates gene expression *via* phosphorylation of RNA polymerase II	↑	↑	↑	↑		(24–27)
CDK9	Facilitates transcriptional elongation *via* phosphorylation of RNA polymerase II	↑	↑	NS*	↑		(28–30)
CDK10	Phosphorylation of ETS2 resulting in ETS2 degradation	↑	↓	↓	N/A		(31–33)
CDK11	Involved in regulation of pre-mRNA splicing	NS*	NS*	↑*	↑*	↑esophageal	(34, 35)
CDK12	Regulates gene expression *via* phosphorylation of RNA polymerase II	NS*	↓↑	NS*	NS*		(36, 37)
CDK13	Involved in transcription regulation and pre-mRNA splicing	↑	↓*	↑	NS*		(38)
CDK14	Activator of Wnt signaling pathway	↑	↑	↑	↑	↑esophageal	(39–43)
CDK15	Inhibits TRAIL-induced apoptosis *via* phosphorylation of survivin	↑*	↓*	NS*	NS*		(44)
CDK16	Promotes skeletal myogenesis and spermatogenesis	NS*	NS*	↑	NS*		(45–47)
CDK17	Involved in neuronal differentiation	↑*	NS*	NS*	NS*		(48)
CDK18	Prevents accumulation of DNA damage and genomic instability	↓*	↑	↓*	↓*		(49, 50)
CDK19	Involved in transcriptional regulation of RNA polymerase II	↓*	↑	NS*	NS*		(44, 51)
CDK20	Promotes transition from G1 to S phase *via* phosphorylation of CDK2	↑	NS*	↑	↓*		(52, 53)

¥ includes hepatocellular and cholangiocarcinoma; ↑ increase in tumor vs. normal tissue; ↓ decrease in tumor vs. normal tissue; NS not significant; * based on Human Protein Atlas (54).

CDKs are generally categorized into two groups, based on their functions (1): cell cycle regulators; and (2) transcription regulators. CDKs involved in cell cycle regulation include CDK1, CDK2, CDK4 and CDK6. These CDKs are regulated through oscillation of expression throughout the cell cycle (55). CDK1 triggers the G2/M phase transition, while CDK2, CDK4 and CDK6 are regulators of the G1/S phase transition (56). CDKs involved in transcription regulation include CDK7, CDK8, CDK9, CDK10 and CDK11. Expression of CDKs involved in transcription regulation do not oscillate and are instead regulated by protein-protein interactions (56). They regulate transcription through phosphorylation of RNA polymerase II and through pre-mRNA splicing regulation.

CDKs often are dysregulated in malignancies, as shown in **Table 1**, causing dysregulation to cell cycle and transcription, leading to abnormal cell proliferation and inhibition of cell death (56). Genetic aberrations of CDKs and cyclins in tumor cells result in continuous cell proliferation or unscheduled cell cycle progression (56). Given their dysregulation in cancer, and their roles in mediating cell cycle progression, CDKs have been considered viable therapeutic targets for cancers, including gastrointestinal cancers.

Cyclin-dependent kinase 10 (CDK10) is a Cdc2-related kinase that was discovered based on its homology to the Cdc2 PSTA1RE amino acid domain (57). CDK10 plays a pivotal role in the regulation of fundamental cellular processes, including cell proliferation, transcription regulation and cell cycle regulation. Initial reports have indicated that CDK10 may act as a tumor suppressor in breast cancer. CDK10 is significantly downregulated in breast cancer compared to normal breast tissue (58). Additionally, CDK10 expression was inversely correlated with tumor stage and lymph node metastasis (58). Importantly, CDK10 expression was associated with better overall survival and may be a predictor of prognosis in breast cancer (58). Additional studies have demonstrated tumor suppressive and oncogenic roles for CDK10 in other malignancies. Specifically, CDK10 has been identified as a candidate tumor suppressor in hepatobiliary cancers, gastric cancer, glioma and nasopharyngeal carcinoma (31, 32, 59–61). Additionally, CDK10 has been shown to promote tumorigenesis in colorectal cancer (CRC) (62). Herein, we present a review of CDK10: its interacting partners, its role in gastrointestinal malignancies and its viability as a therapeutic target.

### Molecular Genetics of CDK10

CDK10 was discovered in 1994 based on its homology to the Cdc2 PSTA1RE amino acid domain. PCR-based cloning was used to amplify cDNA encoding a novel human Cdc2-related kinase, which was called PISSLRE, and later termed CDK10 (57). Amino acid analysis revealed 38-45% identity with other CDKs (57). The chromosomal location of the gene encoding CDK10 was determined to be 16q24.3. The gene *CDK10* (as designed by HUGO) consists of thirteen exons, distributed over approximately 15,000 kilobases of genomic DNA.

**Figure 1:**
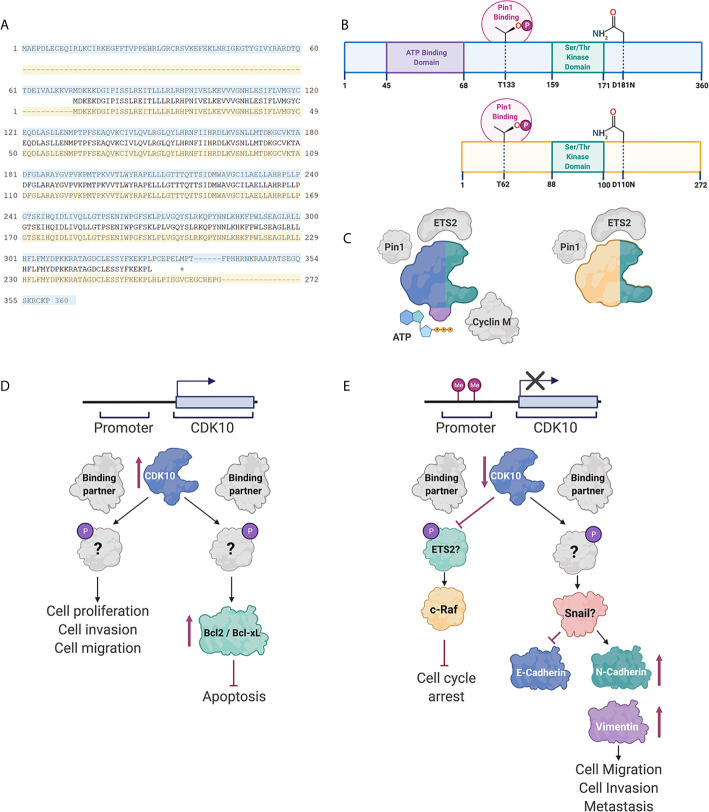
CDK10 isoforms and interacting partners. **(A)** Protein sequence alignment of the full length CDK10 isoform (blue) and the splice variant (yellow). **(B)** Schematics of the full length CDK10 isoform and splice isoform, showing the ATP binding domain, the Ser/Thr kinase domain and Thr133, which is involved in Pin1 binding. **(C)** Models of CDK10 full-length protein and splice isoform with putative binding partners. Figures were created using BioRender. **(D)** Proposed CDK10 oncogenic signaling pathways. **(E)** Proposed CDK10 tumor suppressive signaling pathways. “?” denotes not yet shown in gastrointestinal and hepatobiliary cancers (60, 62–64).

A putative method of regulation of CDK10 is through alternative splicing of pre-mRNA transcripts. Several *CDK10* alternatively spliced isoforms have been identified. These alternatively spliced transcripts differ in exon 11 and in their 5’ and 3’ untranslated regions (UTRs). Of the differentially spliced transcripts identified, two produce functional proteins: the full-length transcript, which encodes for a 360 amino acid protein and a second transcript that encodes a truncated 272 amino acid variant, as shown in **Figure 1**. The latter protein is missing the ATP-binding domain and is therefore enzymatically inactive. Additionally, the shorter isoform does not interact with ETS2 and only weakly interacts with Cyclin M (33). It is therefore thought that alternative splicing is an important method of regulating CDK10 kinase activity.

### Interacting Partners of CDK10

Binding of a CDK to its partner cyclin is generally required for its activation and subsequent kinase function. CDK10 stood alone as the last CDK lacking identification of a cyclin partner. Recently, cyclin M was identified as a CDK10 binding partner by yeast two-hybrid screening and immunoprecipitation (33). The binding of CDK10 to cyclin M is independent of the kinase domain, however, the interaction of CDK10 with cyclin M was shown to regulate the kinase activity of CDK10, in STAR syndrome, (**Figure 1**) (33). This interaction, however, has not been shown in cancers involving the GI tract or hepatobiliary system.

Using a yeast interaction trap, CDK10 was shown to bind to transcription factor ETS2, both *in vitro* and *in vivo*, using human embryonic kidney 293 cells (**Figure 1**) (65). This interaction occurs *via* the N-terminus of ETS2, and mutation of the kinase domain of CDK10 (resulting in CDK10DN variant) did not impact the binding of ETS2 to CDK10. CDK10 has been shown to regulate transactivation of ETS2. Mass spectrometric analysis revealed ETS2 as a substrate for CDK10/cyclin M phosphorylation (33) and it has been reported that phosphorylation of ETS2 by CDK10/Cyclin M results in inhibition of ETS2 transactivation.

Subcellular localization of CDK10 and Cyclin M at the base of the primary cilia, and specific co-localization with centrosomal proteins, suggests a role for CDK10/Cyclin M in ciliogenesis (33). Knockdown of CDK10/Cyclin M significantly decreases stress fiber formation and ciliogenesis in human telomerase reverse transcriptase retinal pigmented epithelial (hTERT RPE-1) cells (33). Using an *in vitro* kinase assay, known core centrosomal proteins and regulators of ciliogenesis and actin dynamics were screened as candidate substrates for CDK10/Cyclin M. This method was used to identify protein kinase C-like 2 (PKN2) as an interacting partner and substrate for CDK10/Cyclin M, both *in vitro* and *in vivo* (33). CDK10/Cyclin M was shown to phosphorylate residues T121 and T124 of PKN2 and furthermore, to repress ciliogenesis in a RhoA-dependent manner. Cyclin M, however, has yet to be shown to be a CDK10 binding partner in gastrointestinal and hepatobiliary cancers.

Pin1 interaction with CDK10 has been documented in ER-positive breast cancer cells (**Figure 1**) (63). Pin1 is known to interact with Ser/Thr-Pro motifs, where the serine or threonine preceding the proline is phosphorylated. CDK10 does not contain any Ser-Pro motifs, however, does contain three Thr-Pro motifs. Mutagenesis of the three Thr-Pro motifs in CDK10 revealed Thr133 as an important residue for Pin1/CDK10 binding. Furthermore, treatment of CDK10 with a phosphatase demonstrated that Pin1 interacts with CDK10 in a phosphorylation-dependent manner. Consequently, interaction of Pin1 with CDK10 results in ubiquitination of CDK10 and subsequent degradation.

There is also evidence of CDK10 interaction with additional binding partners in non-human cell types. Specifically, CDK10 was shown to interact with Hsc70, Hsp90 and EcRB1 in *Helicoverpa armigera* (66). The interaction between CDK10 and Hsc70 and Hsp90 was augmented upon CDK10 phosphorylation. CDK10 forms a complex with Hsc70 and Hsp90, which sequentially binds with EcRB1 to facilitate the interaction between EcRB1 and EcRE to regulate 20E-mediated gene expression (66). Further studies are warranted to assess these as candidate binding partners for CDK10 in humans, and specifically, cancers involving the gastrointestinal tract and hepatobiliary system.

## CDK10 as a Tumor Suppressor in Gastrointestinal and Hepatobiliary Cancers

### Hepatobiliary Cancer

CDK10 has been identified as a candidate tumor suppressor in hepatobiliary cancers, including hepatocellular carcinoma (HCC) and biliary tract cancers (BTC) (31, 32, 64, 67). Examination of HCC tumor tissue revealed decreased expression of *CDK10* mRNA and CDK10 protein compared to adjacent normal liver tissue (31). Immunohistological staining of CDK10 showed weak or no staining in HCC tissue samples. Significantly, CDK10 abundance was found to be inversely correlated to tumor size and tumor stage in HCC. In BTC, downregulation of *CDK10* gene expression and CDK10 protein was observed in cancer tissue and cell lines, and was adversely associated with tumor stage, and lymph node invasion (64). Specifically, CDK10 was significantly downregulated in intrahepatic cholangiocarcinoma and gallbladder cancer, compared to normal tissue.

Several studies have characterized the effects of CDK10 on proliferation of hepatobiliary cancers *in vitro* (31, 64). Zhong et al. found that ectopic expression of *CDK10* in the HCC cell line SMMC-7721 resulted in inhibition of cell proliferation (31). Similarly, in BTC, ectopic expression of *CDK10* decreased cell proliferation, and downregulation of *CDK10* expression significantly increased cell proliferation (64). Furthermore, cell cycle analysis of HCC cells following CDK10 overexpression revealed a significant increase in the G_0_-G_1_ phase population of cells, and a decrease in the S phase population (31). Consistently, BTC cells overexpressing CDK10 had a significant increase in the population of cells in G_1_ phase, and a significant decrease in the population of cells in G_2_/M phase (64).

Studies have also assessed the impact of CDK10 expression on cell invasion and migration (31, 64). In HCC, ectopic expression of *CDK10* significantly delayed wound healing (31). Yu et al. also reported a significant decrease in BTC cell migration upon CDK10 overexpression (64). Consistently, they also reported a significant increase in BTC cell migration following downregulation of CDK10 (64). These studies, however, did not examine the mechanism by which CDK10 inhibits cancer cell invasion and migration. In glioma, CDK10 was shown to regulate cell motility through inhibition of epithelial to mesenchymal transition (EMT) (60). *CDK10* knockdown decreased E-cadherin and increased vimentin and N-cadherin expression. Conversely, overexpression of CDK10 increased E-cadherin and decreased vimentin and N-cadherin expression. CDK10 was shown to regulate expression of the EMT transcription factor, Snail, and the effects of CDK10 on EMT in glioma were partially reversed by manipulation of Snail expression (60). Importantly, Snail is a known regulator of EMT in hepatobiliary cancersCDK10 may regulate cell motility of hepatobiliary cancer cells through inhibition of EMT *via* Snail downregulation.

Therapeutic resistance is a major hindrance on treatment of gastrointestinal cancers. There is significant evident identifying CDK10 as an important modulator of tamoxifen sensitivity in breast cancer, suggesting a potential role for CDK10 in chemosensitivity in other cancers (63, 68). In patients with ER-positive breast cancer, low CDK10 expression was associated with shorter overall survival and clinical resistance to tamoxifen (68). This study used gene silencing to identify CDK10 as a modulator of tamoxifen resistance in breast cancer through regulation of p42/p44 MAPK pathway (68). In BTC, Yu et al. found that knockdown of *CDK10* significantly decreased sensitivity to 5-fluorouracil (64). Furthermore, overexpression of *CDK10* increased BTC cell sensitivity to 5-fluorouracil, adriamycin/epirubicin, cisplatin and hydroxylcamptothecin *in vitro* (64). Similar to endocrine resistance in breast cancer, cell cycle arrest at G_1_ phase was observed in 5-fluorouracil-treated cells overexpressing CDK10 (64, 68). In HCC, overexpression of CDK10 increased chemosensitivity to cisplatin and epirubicin in SMMC-7721 cells but not HepG2 cells (31). Furthermore, overexpression of CDK10 increased gall bladder cancer sensitivity to gemcitabine (69). Anticipating a mechanism similar to endocrine therapy resistance in breast cancer, CDK10 expression was shown to downregulate c-Raf levels in BTC (64, 69). Furthermore, in gall bladder cancer, knockdown of c-Raf resulted in a significant increase in gemcitabine sensitivity in cells overexpressing CDK10 (69). Downregulation of CDK10 increased ETS2-mediated transcription of c-Raf, resulting in activation of the MAPK pathway. Additionally, Khanal et al. investigated the association between CDK10 and Pin1 expression in tamoxifen-resistant breast cancer cells (63). This study observed a significant inverse correlation between Pin1 and CDK10 expression in tamoxifen-resistant breast cancer. Khanal et al. also found that overexpression of CDK10 increased breast cancer cell sensitivity to tamoxifen treatment, and decreased Pin1-mediated c-Raf phosphorylation (63). However, further research is warranted to delineate the mechanism by which CDK10 regulates c-Raf levels in gastrointestinal cancers and how that results in cell cycle arrest.

### Gastric Cancer

In addition to hepatobiliary cancers, CDK10 has been identified as a candidate tumor suppressor in gastric cancer. Independent studies found decreased expression of CDK10 in gastric cancer compared to normal gastric tissue (32, 67). Consistently, these studies found a significant correlation between loss of CDK10 expression and advanced tumor stage, lymph node invasion and distant metastasis, in patients with gastric cancer. Furthermore, these studies also identified loss of CDK10 expression as an unfavorable prognostic marker in gastric cancer. However, Fukui et al. found that CDK10 was upregulated in peritoneal and liver metastases in human gastric cancer cell lines established following injection into nude mice (70).

Studies have also assessed the effect of CDK10 on cell proliferation and cell motility in gastric cancers. Ectopic expression of *CDK10* decreased cell proliferation, while downregulation of *CDK10* expression significantly increased cell proliferation (32). Treatment of gastric cancer cells with quercetin, a flavonol shown to induce apoptosis, significantly decreased expression of *CDK10* (71). Additionally, in gastric cancer, overexpression of CDK10 decreased cell invasion, while knockdown of CDK10 promoted cell invasion (32).

### Mechanisms of CDK10 Downregulation in Gastrointestinal Cancers

As previously mentioned, CDK10 expression is downregulated in hepatobiliary cancers and gastric cancers (31, 32, 64). Mechanisms of CDK10 downregulation have not been examined in gastrointestinal cancers. Chromosomal deletions at the q24 region of chromosome 16 are associated with human cancers, including gastric cancer and HCC (72, 73). Furthermore, aberrant methylation of chromosome 16 is a mechanism of gene expression dysregulation in chronic hepatitis, liver cirrhosis and HCC (74, 75). Promoter hypermethylation was found to be a mechanism of CDK10 suppression in breast cancer and nasopharyngeal carcinoma (61, 68). Therefore, suppression of CDK10 may result from loss of heterozygosity and hypermethylation at the q24 region of chromosome 16. Further research is warranted to assess loss of heterozygosity and promoter hypermethylation as potential mechanisms of CDK10 downregulation in gastrointestinal cancers.

## CDK10 in Colorectal Cancer

A meta-analysis of CRC gene expression profiling studies identified *CDK10* as a gene consistently upregulated in CRC (76). Consistent with this meta-analysis, Weiswald et al. found overexpression of CDK10 in CRC tissue and CRC cell lines, compared to matched normal tissue and normal colon cells, respectively (62). This observation is similar to other studies that found upregulation of *CDK10* in prostate cancer and seminomas (77, 78). Furthermore, increased expression of CDK10 was found to be correlated with lymph node positive tumors in CRC (79).

Weiswald et al. also found that CRC growth and survival were significantly affected by manipulation of *CDK10* gene expression. Specifically, CDK10 knockdown decreased cell survival and promoted apoptosis in CRC cell lines. Similarly, overexpression of CDK10 increased cell proliferation and inhibited apoptosis *in vitro*, in a Bcl-2/Bcl-_XL_-dependent manner. Consistently, suppression of CDK10 in patient-derived xenograft CRC tumors inhibited tumor growth and decreased expression of Bcl-2 *in vivo*. Interestingly, CDK10 expression did not affect cell cycle progression in CRC, indicating that the impact of CDK10 on cell proliferation and apoptosis is independent of cell cycle regulation.

Furthermore, Weiswald et al. evaluated the effects of CDK10 on chemotherapy resistance. CRC cell lines overexpressing CDK10 were significantly less responsive to 5-fluorouracil compared to controls (62). Additionally, compared to CDK10WT, cell lines expressing CDK10DN were more sensitive to 5-fluorouracil, indicating kinase involvement in CDK10-mediated chemoresistance in CRC. Indeed, CDK10 expression may be a viable biomarker for chemotherapy resistance and should be assessed as a potential biomarker for CRC recurrence. Importantly, while this study suggests a kinase-dependent role for CDK10, an interacting partner to CDK10 has yet to be identified and implicated in CRC.

The role of CDK10 in CRC cell invasion and migration is yet to be determined. However, Zehra et al. demonstrated upregulation of CDK10 and ETS2 in a corneal epithelial wound healing model (80). This study infers a potential role for CDK10 in cancer metastasis, as inhibition of CDK10 resulted in a significant delay in corneal epithelial cell migration.

## CDK10 as a Therapeutic Target for Gastrointestinal Cancers

Research has been dedicated to the development of candidate CDK small molecule inhibitors, however, the majority of CDK inhibitors lack specificity and clinical trials have had disappointing outcomes. Several strategies have been employed in drug discovery to develop effective CDK inhibitors, including reversible ATP-competitive and non-competitive inhibition, reversible and irreversible allosteric inhibition, antibodies, and CDK-targeted degradation. Non-specific pan-CDK inhibitors exhibit low anti-cancer activity and high toxicity. Due to their non-specificity, these inhibitors block several cell processes, including cell proliferation, transcription, and translation. Given the tumor suppressive nature of CDK10 in breast cancer, HCC, BTC and gastric cancer, targeting CDK10 *via* pan-CDK inhibitors may have limited the therapeutic response.

The development of therapeutics targeting CDK10 has been hindered by lack of a CDK10 activity assay, and lack of a specific small molecule inhibitor to identify novel therapies. Discovery of a small molecule inhibitor for CDK10 would facilitate further exploration of its biological functions and affirm its candidacy as a therapeutic target, specifically for CRC. Recently, Robert et al. described a novel CDK10/Cyclin M *in vitro* activity assay (81). This luminescence-based assay uses a synthetic peptide phosphorylation substrate for the CDK10/Cyclin M complex.

Flavopiridol is the most extensively studied pan-CDK inhibitor. It has been shown to inhibit CDK1, CDK2, CDK4, CDK6, CDK9 and CDK10. While flavopiridol can inhibit CDK10, the IC50 for flavopiridol inhibition against CDK10/Cyclin M is less potent than flavopiridol inhibition of other CDKs (81). *In vitro* studies in gastrointestinal cells demonstrated that flavopiridol was effective in inducing apoptosis through blockage of cell cycle progression at G_1_ (82, 83). However, clinical trials in patients with gastrointestinal cancers did not result in favorable outcomes (84, 85) and reported significant toxicity among patients.

Additional CDK inhibitors such as dinaciclib, SDS-032, AZD4573, AT7519 and riviciclib were all tested on CDK10/Cyclin M, however none of these potently inhibited CDK10 kinase activity (81). Ibrahim et al. synthesized novel flavopiridol analogs and assessed their inhibitory activity on CDK2, CDK5 and CDK9 (86). This series of inhibitors was more potent towards CDK9 than the other CDKs examined in the study. Given the close relation between CDK9 and CDK10, these inhibitors may exhibit inhibitory activity towards CDK10 (2).

The development of therapeutics targeting CDK10 should account for the tissue-specific biological activity of CDK10. Given that CDK10 acts as a tumor suppressor in some gastrointestinal and hepatobiliary cancers, future drug development should focus on inhibiting other CDKs, while maintaining activity of CDK10. In these cancers, CDK10 expression levels may be indicative of chemoresistance. The promotion of tumorigenesis by CDK10 in CRC suggests its inhibition is a promising therapeutic strategy. Given that the kinase domain has been implicated CDK10-mediated inhibition of apoptosis in CRC, inhibition of CDK10 kinase activity may be an effective therapeutic approach. The development of a CDK10-specific inhibitor may be a viable therapeutic target for the treatment of CRC.

## Conclusion

CDK10 has been implicated as both a tumor suppressor and an oncogene in gastrointestinal and hepatobiliary cancers. CDK10 is involved in cell proliferation, cell motility, and plays an important role in chemosensitivity and chemoresistance. Further studies are warranted to understand the tissue-specific functions of CDK10 and the mechanisms that influence its oncogenic and tumor suppressive potential in gastrointestinal cancer. Implications of CDK10 as an oncogene in CRC make inhibition of CDK10 a viable therapeutic strategy. The development of therapeutics targeting CDK10 has been hindered by lack of a high throughput CDK10 activity screening assay. Detection of CDK10 kinase activity will allow for identification of small molecule inhibitors of CDK10. This will aid in further understanding the role of CDK10 in disease progression, and the development of therapeutics for the treatment of gastrointestinal cancers.

## Author Contributions

ZB: drafting and editing the manuscript. IT: editing and critically reading manuscript. All authors contributed to the article and approved the submitted version.

## Funding

This study was supported by the Canadian Institutes of Health Research (CIHR) (Grant #MOP-82881) and CIHR New Investigator salary award to IT (MSH-95344).

## Conflict of Interest

The authors declare that the research was conducted in the absence of any commercial or financial relationships that could be construed as a potential conflict of interest.

## References

[1] Lim S Kaldis P. Cdks, Cyclins and CKIs: Roles Beyond Cell Cycle Regulation. Development (2013) 140:3079–93. 10.1242/dev.091744 23861057

[2] Cao L Chen F Yang X Xu W Xie J Yu L. Phylogenetic Analysis of CDK and Cyclin Proteins in Premetazoan Lineages. BMC Evol Biol (2014) 14:10. 10.1186/1471-2148-14-10 24433236 PMC3923393

[3] Piao J Zhu L Sun J Li N Dong B Yang Y. High Expression of CDK1 and BUB1 Predicts Poor Prognosis of Pancreatic Ductal Adenocarcinoma. Gene (2019) 701:15–22. 10.1016/j.gene.2019.02.081 30898709

[4] Gan W Zhao H Li T Liu K Huang J. CDK1 Interacts With iASPP to Regulate Colorectal Cancer Cell Proliferation Through p53 Pathway. Oncotarget (2017) 8:71618–29. 10.18632/oncotarget.17794 PMC5641076 29069733

[5] Shi Q Ni X Lei M Xia Q Dong Y Zhang Q. Phosphorylation of Islet-1 Serine 269 by CDK1 Increases its Transcriptional Activity and Promotes Cell Proliferation in Gastric Cancer. Mol Med (2021) 27:47. 10.1186/s10020-021-00302-6 33962568 PMC8106192

[6] Yang W-X Pan Y-Y You C-G. Cdk1, CCNB1, Cdc20, BUB1, Mad2l1, MCM3, Bub1b, MCM2, and RFC4 may Be Potential Therapeutic Targets for Hepatocellular Carcinoma Using Integrated Bioinformatic Analysis. BioMed Res Int (2019) 2019:1245072. 10.1155/2019/1245072 31737652 PMC6815605

[7] Li J-Q Miki H Ohmori M Wu F Funamoto Y. Expression of Cyclin E and Cyclin-Dependent Kinase 2 Correlates With Metastasis and Prognosis in Colorectal Carcinoma. Hum Pathol (2001) 32:945–53. 10.1053/hupa.2001.27116 11567224

[8] Ren S Rollins BJ. Cyclin C/Cdk3 Promotes Rb-Dependent G0 Exit. Cell (2004) 117:239–51. 10.1016/S0092-8674(04)00300-9 15084261

[9] Lu J Zhang ZL Huang D Tang N Li Y Peng Z. Cdk3-promoted Epithelial-Mesenchymal Transition Through Activating AP-1 is Involved in Colorectal Cancer Metastasis. Oncotarget (2016) 7:7012–28. 10.18632/oncotarget.6875 PMC4872765 26755651

[10] Lu JW Lin YM Chang JG Yeh KT Chen RM Tsai JJ. Clinical Implications of Deregulated CDK4 and Cyclin D1 Expression in Patients With Human Hepatocellular Carcinoma. Med Oncol (2013) 30:379. 10.1007/s12032-012-0379-5 23292829

[11] Lindberg D Hessman O Åkerström G Westin G. Cyclin-Dependent Kinase 4 (Cdk4) Expression in Pancreatic Endocrine Tumors. Neuroendocrinology (2007) 86:112–8. 10.1159/000106762 17664862

[12] Wang S Wang X Gao Y Peng Y Dong N Xie Q. RN181 is a Tumor Suppressor in Gastric Cancer by Regulation of the ERK/MAPK-cyclin D1/CDK4 Pathway. J Pathol (2019) 248:204–16. 10.1002/path.5246 PMC6593865 30714150

[13] Ikeda K Monden T Tsujie M Izawa H Yamamoto H Ohnishi T. [Cyclin D, CDK4 and p16 Expression in Colorectal Cancer]. Nihon Rinsho (1996) 54:1054–9. 8920673

[14] Zhuang K Zhang J Xiong M Wang X Luo X Han L. CDK5 Functions as a Tumor Promoter in Human Colorectal Cancer Via Modulating the ERK5–AP-1 Axis. Cell Death Dis (2016) 7:e2415–5. 10.1038/cddis.2016.333 PMC5133995 27735944

[15] Ohshima T Ward JM Huh CG Longenecker G Veeranna Pant HC. Targeted Disruption of the Cyclin-Dependent Kinase 5 Gene Results in Abnormal Corticogenesis, Neuronal Pathology and Perinatal Death. Proc Natl Acad Sci (1996) 93:11173–8. 10.1073/pnas.93.20.11173 PMC38303 8855328

[16] Zhang R Lin P Yang H He Y Dang Y-W Feng Z-B. Clinical Role and Biological Function of CDK5 in Hepatocellular Carcinoma: A Study Based on Immunohistochemistry, RNA-seq and *In Vitro* Investigation. Oncotarget (2017) 8:108333–54. 10.18632/oncotarget.22659 PMC5752448 29312535

[17] Eggers JP Grandgenett PM Collisson EC Lewallen ME Tremayne J Singh PK. Cyclin-Dependent Kinase 5 Is Amplified and Overexpressed in Pancreatic Cancer and Activated by Mutant K-Ras. Clin Cancer Res (2011) 17:6140–50. 10.1158/1078-0432.CCR-10-2288 PMC3425449 21825040

[18] Cao L Zhou J Zhang J Wu S Yang X Zhao X. Cyclin-Dependent Kinase 5 Decreases in Gastric Cancer and Its Nuclear Accumulation Suppresses Gastric Tumorigenesis. Clin Cancer Res (2015) 21:1419–28. 10.1158/1078-0432.CCR-14-1950 25609066

[19] Feng L Xie Y Zhang H Wu Y. miR-107 Targets Cyclin-Dependent Kinase 6 Expression, Induces Cell Cycle G1 Arrest and Inhibits Invasion in Gastric Cancer Cells. Med Oncol (2012) 29:856–63. 10.1007/s12032-011-9823-1 21264532

[20] Tadano T Kakuta Y Hamada S Shimodaira Y Kuroha M Kawakami Y. MicroRNA-320 Family is Downregulated in Colorectal Adenoma and Affects Tumor Proliferation by Targeting CDK6. World J gastrointestinal Oncol (2016) 8:532–42. 10.4251/wjgo.v8.i7.532 PMC4942741 27559432

[21] Baba Y Watanabe M Murata A Shigaki H Miyake K Ishimoto T. : LINE-1 Hypomethylation, DNA Copy Number Alterations, and CDK6 Amplification in Esophageal Squamous Cell Carcinoma. Clin Cancer Res (2014) 20:1114–24. 10.1158/1078-0432.CCR-13-1645 24423610

[22] Lu P Geng J Zhang L Wang Y Niu N Fang Y. THZ1 Reveals CDK7-dependent Transcriptional Addictions in Pancreatic Cancer. Oncogene (2019) 38:3932–45. 10.1038/s41388-019-0701-1 30692639

[23] Wang Q Li M Zhang X Huang H Huang J Ke J. Upregulation of CDK7 in Gastric Cancer Cell Promotes Tumor Cell Proliferation and Predicts Poor Prognosis. Exp Mol Pathol (2016) 100:514–21. 10.1016/j.yexmp.2016.05.001 27155449

[24] Xu W Wang Z Zhang W Qian K Li H Kong D. Mutated K-ras Activates CDK8 to Stimulate the Epithelial-to-Mesenchymal Transition in Pancreatic Cancer in Part Via the Wnt/β-Catenin Signaling Pathway. Cancer Lett (2015) 356:613–27. 10.1016/j.canlet.2014.10.008 25305448

[25] Seo JO Han SI Lim SC. Role of CDK8 and Beta-Catenin in Colorectal Adenocarcinoma. Oncol Rep (2010) 24:285–91. 10.3892/or_00000858 20514474

[26] Kim M-Y Han SI Lim S-C. Roles of Cyclin-Dependent Kinase 8 and β-Catenin in the Oncogenesis and Progression of Gastric Adenocarcinoma. Int J Oncol (2011) 38:1375–83. 10.3892/ijo.2011.948 21344156

[27] Han SI Lim S-C. Expression and Prognostic Significance of CDK8 and β-Catenin in Hepatocellular Carcinoma. In Vivo (Athens Greece) (2020) 34:1387–94. 10.21873/invivo.11918 PMC7279855 32354935

[28] Kretz AL Schaum M Richter J Kitzig EF Engler CC Leithäuser F. CDK9 is a Prognostic Marker and Therapeutic Target in Pancreatic Cancer. Tumor Biol (2017) 39:1010428317694304. 10.1177/1010428317694304 28231737

[29] Lu Y Tang L Zhang Q Zhang Z Wei W. MicroRNA-613 Inhibits the Progression of Gastric Cancer by Targeting CDK9. Artif Cells Nanomed Biotechnol (2018) 46:980–4. 10.1080/21691401.2017.1351983 28701053

[30] Wang J Liu J Tian F Zhan Y Kong D. Cyclin-Dependent Kinase 9 Expression and its Association With CD8(+) T Cell Infiltration in Microsatellite-Stable Colorectal Cancer. Oncol Lett (2019) 18:6046–56. 10.3892/ol.2019.10970 PMC6865572 31788079

[31] Zhong XY Xu XX Yu JH Jiang GX Yu Y Tai S. Clinical and Biological Significance of Cdk10 in Hepatocellular Carcinoma. Gene (2012) 498:68–74. 10.1016/j.gene.2012.01.022 22326270

[32] You Y Bai F Ye Z Zhang N Yao L Tang Y. Downregulated CDK10 Expression in Gastric Cancer: Association With Tumor Progression and Poor Prognosis. Mol Med Rep (2018) 17:6812–8. 10.3892/mmr.2018.8662 29512714

[33] Guen VJ Gamble C Flajolet M Unger S Thollet A Ferandin Y. CDK10/Cyclin M is a Protein Kinase That Controls ETS2 Degradation and is Deficient in STAR Syndrome. Proc Natl Acad Sci USA (2013) 110:19525–30. 10.1073/pnas.1306814110 PMC3845122 24218572

[34] Hu D Mayeda A Trembley JH Lahti JM Kidd VJ. CDK11 Complexes Promote pre-mRNA Splicing. J Biol Chem (2003) 278:8623–9. 10.1074/jbc.M210057200 12501247

[35] Du Y Yan D Yuan Y Xu J Wang S Yang Z. Cdk11(p110) Plays a Critical Role in the Tumorigenicity of Esophageal Squamous Cell Carcinoma Cells and is a Potential Drug Target. Cell Cycle (Georgetown Tex) (2019) 18:452–66. 10.1080/15384101.2019.1577665 PMC6422471 30722725

[36] Liu M Fan H Li T Sihong L Qiao S Bi J. Low Expression of CDK12 in Gastric Cancer is Correlated With Advanced Stage and Poor Outcome. Pathol - Res Pract (2020) 216:152962. 10.1016/j.prp.2020.152962 32534699

[37] Ji J Zhou C Wu J Cai Q Shi M Zhang H. Expression Pattern of CDK12 Protein in Gastric Cancer and its Positive Correlation With CD8(+) Cell Density and CCL12 Expression. Int J Med Sci (2019) 16:1142–8. 10.7150/ijms.34541 PMC6743279 31523177

[38] Kim H-E Kim D-G Lee KJ Son JG Song M-Y Park Y-M. Frequent Amplification of CENPF, GMNN and CDK13 Genes in Hepatocellular Carcinomas. PloS One (2012) 7:e43223. 10.1371/journal.pone.0043223 22912832 PMC3418236

[39] Chen L Wang Y Jiang W Ni R Wang Y Ni S. CDK14 Involvement in Proliferation Migration and Invasion of Esophageal Cancer. Ann Trans Med (2019) 7:681–1. 10.21037/atm.2019.11.105 PMC6944537 31930082

[40] Sun Y Zhu Q Yang W Shan Y Yu Z Zhang Q. Lncrna H19/miR-194/PFTK1 Axis Modulates the Cell Proliferation and Migration of Pancreatic Cancer. J Cell Biochem (2019) 120:3874–86. 10.1002/jcb.27669 30474270

[41] Yang L Zhu J Huang H Yang Q Cai J Wang Q. Pftk1 Promotes Gastric Cancer Progression by Regulating Proliferation, Migration and Invasion. PloS One (2015) 10:e0140451. 10.1371/journal.pone.0140451 26488471 PMC4619205

[42] Pang EY Bai AH To KF Sy SM Wong NL Lai PB. Identification of PFTAIRE Protein Kinase 1, a Novel Cell Division Cycle-2 Related Gene, in the Motile Phenotype of Hepatocellular Carcinoma Cells. Hepatology (2007) 46:436–45. 10.1002/hep.21691 17559150

[43] Mao Y Jia Y Zhu H Wang W Jin Q Huang F. High Expression of PFTK1 in Cancer Cells Predicts Poor Prognosis in Colorectal Cancer. Mol Med Rep (2017) 16:224–30. 10.3892/mmr.2017.6560 28498444

[44] Park MH Kim SY Kim YJ Chung Y-H. ALS2CR7 (CDK15) Attenuates TRAIL Induced Apoptosis by Inducing Phosphorylation of Survivin Thr34. Biochem Biophys Res Commun (2014) 450:129–34. 10.1016/j.bbrc.2014.05.070 24866247

[45] Mikolcevic P Sigl R Rauch V Hess MW Pfaller K Barisic M. Cyclin-Dependent Kinase 16/PCTAIRE Kinase 1 is Activated by Cyclin Y and is Essential for Spermatogenesis. Mol Cell Biol (2012) 32:868–79. 10.1128/MCB.06261-11 PMC3272973 22184064

[46] Shimizu K Uematsu A Imai Y Sawasaki T. Pctaire1/Cdk16 Promotes Skeletal Myogenesis by Inducing Myoblast Migration and Fusion. FEBS Lett (2014) 588:3030–7. 10.1016/j.febslet.2014.05.060 24931367

[47] Liu Q Wang C Jiang Z Li S Li F Tan H-B. circRNA 001306 Enhances Hepatocellular Carcinoma Growth by Up-Regulating CDK16 Expression Via Sponging Mir-584-5p. J Cell Mol Med (2020) 24:14306–15. 10.1111/jcmm.16047 PMC7754030 33135290

[48] Kerr N Pintzas A Holmes F Hobson SA Pope R Wallace M. The Expression of ELK Transcription Factors in Adult DRG: Novel Isoforms, Antisense Transcripts and Upregulation by Nerve Damage. Mol Cell Neurosci (2010) 44:165–77. 10.1016/j.mcn.2010.03.005 PMC2862884 20304071

[49] Barone G Staples CJ Ganesh A Patterson KW Bryne DP Myers KN. Human CDK18 Promotes Replication Stress Signaling and Genome Stability. Nucleic Acids Res (2016) 44:8772–85. 10.1093/nar/gkw615 PMC5062979 27382066

[50] Li P Ge D Li P Hu F Chu J Chen X. CXXC Finger Protein 4 Inhibits the CDK18-ERK1/2 Axis to Suppress the Immune Escape of Gastric Cancer Cells With Involvement of ELK1/MIR100HG Pathway. J Cell Mol Med (2020) 24:10151–65. 10.1111/jcmm.15625 PMC7520267 32715641

[51] Zhao JQ Li XN Fu LP Zhang N Cai JH. ISOC1 Promotes the Proliferation of Gastric Cancer Cells by Positively Regulating CDK19. Eur Rev Med Pharmacol Sci (2020) 24:11602–9. 10.26355/eurrev_202011_23803 33275227

[52] Feng H Yu Z Tian Y Lee YY Li MS Go MY. A CCRK-EZH2 Epigenetic Circuitry Drives Hepatocarcinogenesis and Associates With Tumor Recurrence and Poor Survival of Patients. J Hepatol (2015) 62:1100–11. 10.1016/j.jhep.2014.11.040 25500144

[53] An X Ng SS Xie D Zeng YX Sze J Wang J. Functional Characterisation of Cell Cycle-Related Kinase (CCRK) in Colorectal Cancer Carcinogenesis. Eur J Cancer (2010) 46:1752–61. 10.1016/j.ejca.2010.04.007 20466538

[54] Uhlén M Björling E Agaton C Szigyarto CA-K Amini B Andersen E. : A Human Protein Atlas for Normal and Cancer Tissues Based on Antibody Proteomics. Mol Cell Proteomics (2005) 4:1920–32. 10.1074/mcp.M500279-MCP200 16127175

[55] Simmons Kovacs LA Mayhew MB Orlando DA Jin Y Li Q Huang C. Cyclin-Dependent Kinases are Regulators and Effectors of Oscillations Driven by a Transcription Factor Network. Mol Cell (2012) 45:669–79. 10.1016/j.molcel.2011.12.033 PMC3578314 22306294

[56] Malumbres M Barbacid M. Cell Cycle, CDKs and Cancer: A Changing Paradigm. Nat Rev Cancer (2009) 9:153–66. 10.1038/nrc2602 19238148

[57] Graña X Claudio PP De Luca A Sang N Giordano A. PISSLRE, a Human Novel CDC2-related Protein Kinase. Oncogene (1994) 9:2097–103. 8208557

[58] You Y Li H Qin X Zhang Y Song W Ran Y. Decreased CDK10 Expression Correlates With Lymph Node Metastasis and Predicts Poor Outcome in Breast Cancer Patients - A Short Report. Cell Oncol (Dordr) (2015) 38:485–91. 10.1007/s13402-015-0246-4 PMC13004193 26392360

[59] Crawford J Ianzano L Savino M Whitmore S Cleton-Jansen AM Settasatian C. The PISSLRE Gene: Structure, Exon Skipping, and Exclusion as Tumor Suppressor in Breast Cancer. Genomics (1999) 56:90–7. 10.1006/geno.1998.5676 10036189

[60] Li H You Y Liu J. Cyclin−Dependent Kinase 10 Prevents Glioma Metastasis Via Modulation of Snail Expression. Mol Med Rep (2018) 18:1165–70. 10.3892/mmr.2018.9059 29845196

[61] You Y Yang W Wang Z Zhu H Li H Lin C. Promoter Hypermethylation Contributes to the Frequent Suppression of the CDK10 Gene in Human Nasopharyngeal Carcinomas. Cell Oncol (Dordr) (2013) 36:323–31. 10.1007/s13402-013-0137-5 PMC13012668 23740091

[62] Weiswald L-B Hasan MR Wong JCT Pasiliao CC Rahman M Ren J. Inactivation of the Kinase Domain of CDK10 Prevents Tumor Growth in a Preclinical Model of Colorectal Cancer, and is Accompanied by Downregulation of Bcl-2. Mol Cancer Ther (2017) 16(10):2292–303. 10.1158/1535-7163.MCT-16-0666 28663269

[63] Khanal P Yun HJ Lim SC Ahn SG Yoon HE Kang KW. Proyl Isomerase Pin1 Facilitates Ubiquitin-Mediated Degradation of Cyclin-Dependent Kinase 10 to Induce Tamoxifen Resistance in Breast Cancer Cells. Oncogene (2012) 31:3845–56. 10.1038/onc.2011.548 22158035

[64] Yu JH Zhong XY Zhang WG Wang ZD Dong Q Tai S. CDK10 Functions as a Tumor Suppressor Gene and Regulates Survivability of Biliary Tract Cancer Cells. Oncol Rep (2012) 27:1266–76. 10.3892/or.2011.1617 PMC3583593 22209942

[65] Kasten M Giordano A. Cdk10, a Cdc2-related Kinase, Associates With the Ets2 Transcription Factor and Modulates its Transactivation Activity. Oncogene (2001) 20:1832–8. 10.1038/sj.onc.1204295 11313931

[66] Liu W Cai M-J Wang J-X Zhao X-F. In a Nongenomic Action, Steroid Hormone 20-Hydroxyecdysone Induces Phosphorylation of Cyclin-Dependent Kinase 10 to Promote Gene Transcription. Endocrinology (2014) 155:1738–50. 10.1210/en.2013-2020 24517229

[67] Zhao BW Chen S Li YF Xiang J Zhou ZW Peng JS. Low Expression of CDK10 Correlates With Adverse Prognosis in Gastric Carcinoma. J Cancer (2017) 8:2907–14. 10.7150/jca.20142 PMC5604441 28928881

[68] Iorns E Turner NC Elliott R Syed N Garrone O Gasco M. Identification of CDK10 as an Important Determinant of Resistance to Endocrine Therapy for Breast Cancer. Cancer Cell (2008) 13:91–104. 10.1016/j.ccr.2008.01.001 18242510

[69] Yu J Zhang W Lu B Qian H Tang H Zhu Z. miR-433 Accelerates Acquired Chemoresistance of Gallbladder Cancer Cells by Targeting Cyclin M. Oncol Lett (2018) 15:3305–12. 10.3892/ol.2017.7708 PMC5778899 29435072

[70] Fukui R Nishimori H Hata F Yasoshima T Ohno K Nomura H. Et Al: Metastases-related Genes in the Classification of Liver and Peritoneal Metastasis in Human Gastric Cancer. J Surg Res (2005) 129:94–100. 10.1016/j.jss.2005.04.030 16054651

[71] Shang HS Lu HF Lee CH Chiang HS Chu YL Chen A. Quercetin Induced Cell Apoptosis and Altered Gene Expression in AGS Human Gastric Cancer Cells. Environ Toxicol (2018) 33:1168–81. 10.1002/tox.22623 30152185

[72] Mori Y Matsunaga M Abe T Fukushige S Miura K Sunamura M. Chromosome Band 16q24 is Frequently Deleted in Human Gastric Cancer. Br J Cancer (1999) 80:556–62. 10.1038/sj.bjc.6690391 PMC2362314 10408866

[73] Riou P Saffroy R Comoy J Gross-Goupil M Thiery JP Emile JF. Investigation in Liver Tissues and Cell Lines of the Transcription of 13 Genes Mapping to the 16q24 Region That are Frequently Deleted in Hepatocellular Carcinoma. Clin Cancer Res (2002) 8:3178–86. 12374686

[74] Kanai Y Ushijima S Tsuda H Sakamoto M Sugimura T Hirohashi S. Aberrant DNA Methylation on Chromosome 16 is an Early Event in Hepatocarcinogenesis. Japanese J Cancer Res Gann (1996) 87:1210–7. 10.1111/j.1349-7006.1996.tb03135.x PMC5921026 9045955

[75] Kanai Y Ushijima S Tsuda H Sakamoto M Hirohashi S. Aberrant DNA Methylation Precedes Loss of Heterozygosity on Chromosome 16 in Chronic Hepatitis and Liver Cirrhosis. Cancer Lett (2000) 148:73–80. 10.1016/S0304-3835(99)00316-X 10680595

[76] Chan SK Griffith OL Tai IT Jones SJ. Meta-Analysis of Colorectal Cancer Gene Expression Profiling Studies Identifies Consistently Reported Candidate Biomarkers. Cancer Epidemiol Biomarkers Prev (2008) 17:543–52. 10.1158/1055-9965.EPI-07-2615 18349271

[77] Magee JA Araki T Patil S Ehrig T True L Humphrey PA. Expression Profiling Reveals Hepsin Overexpression in Prostate Cancer. Cancer Res (2001) 61:5692–6. 11479199

[78] Leman ES Magheli A Yong KM Netto G Hinz S Getzenberg RH. Identification of Nuclear Structural Protein Alterations Associated With Seminomas. J Cell Biochem (2009) 108:1274–9. 10.1002/jcb.22357 19795381

[79] Han SW Ahn JY Lee S Noh YS Jung HC Lee MH. Gene Expression Network Analysis of Lymph Node Involvement in Colon Cancer Identifies AHSA2, CDK10, and CWC22 as Possible Prognostic Markers. Sci Rep (2020) 10:7170. 10.1038/s41598-020-63806-x 32345988 PMC7189385

[80] Zehra M Mushtaq S Ghulam Musharraf S Ghani R Ahmed N. Association of Cyclin Dependent Kinase 10 and Transcription Factor 2 During Human Corneal Epithelial Wound Healing *In Vitro* Model. Sci Rep (2019) 9:11802. 10.1038/s41598-019-48092-6 31413335 PMC6694192

[81] Robert T Johnson JL Guichaoua R Yaron TM Bach S Cantley LC. Development of a CDK10/CycM *In Vitro* Kinase Screening Assay and Identification of First Small-Molecule Inhibitors. Front Chem (2020) 8:147. 10.3389/fchem.2020.00147 32175313 PMC7056863

[82] Jung C Motwani M Kortmansky J Sirotnak FM She Y Gonen M. The Cyclin-Dependent Kinase Inhibitor Flavopiridol Potentiates Gamma-Irradiation-Induced Apoptosis in Colon and Gastric Cancer Cells. Clin Cancer Res (2003) 9:6052–61. 14676132

[83] Miyashita K Shiraki K Fuke H Inoue T Yamanaka Y Yamaguchi Y. The Cyclin-Dependent Kinase Inhibitor Flavopiridol Sensitizes Human Hepatocellular Carcinoma Cells to TRAIL-induced Apoptosis. Int J Mol Med (2006) 18:249–56. 10.3892/ijmm.18.2.249 16820931

[84] Ang C O’Reilly EM Carvajal RD Capanu M Gonen M Doyle L. A Nonrandomized, Phase II Study of Sequential Irinotecan and Flavopiridol in Patients With Advanced Hepatocellular Carcinoma. Gastrointestinal Cancer Res GCR (2012) 5:185–9. PMC3533846 23293699

[85] Aklilu M Kindler HL Donehower RC Mani S Vokes EE. Phase II Study of Flavopiridol in Patients With Advanced Colorectal Cancer. Ann Oncol (2003) 14:1270–3. 10.1093/annonc/mdg343 12881391

[86] Ibrahim N Bonnet P Brion J-D Peyrat J-F Bignon J Levaique H. Identification of a New Series of Flavopiridol-Like Structures as Kinase Inhibitors With High Cytotoxic Potency. Eur J Med Chem (2020) 199:112355. 10.1016/j.ejmech.2020.112355 32402934

